# Supplementary material for: Dynamics of Endophytic Fungal Communities Associated with Cultivated Medicinal Plants in Farmland Ecosystem
Source: J Fungi (Basel). 2023 Dec 4;9(12):1165. doi: 10.3390/jof9121165 (PMC10744690; doi:10.3390/jof9121165)
Supplement: Supplementary file 1 [file jof-09-01165-s001.zip › jof-2675379-supplementary.pdf]

Journal of
Fungi

MDPI

Table S1. Meteorological data of Anguo City, Hebei Province.

	Average Minimum Temperature (°C)	Average Maximum Temperature (°C)	Average Humidity (%)	Average Precipitation (mm)
In May 2021	13	27	54	20.3
In June 2021	19	31	63	25.5
In October 2021	6	18	79	12.2
In November 2021	0	12	67	6.1

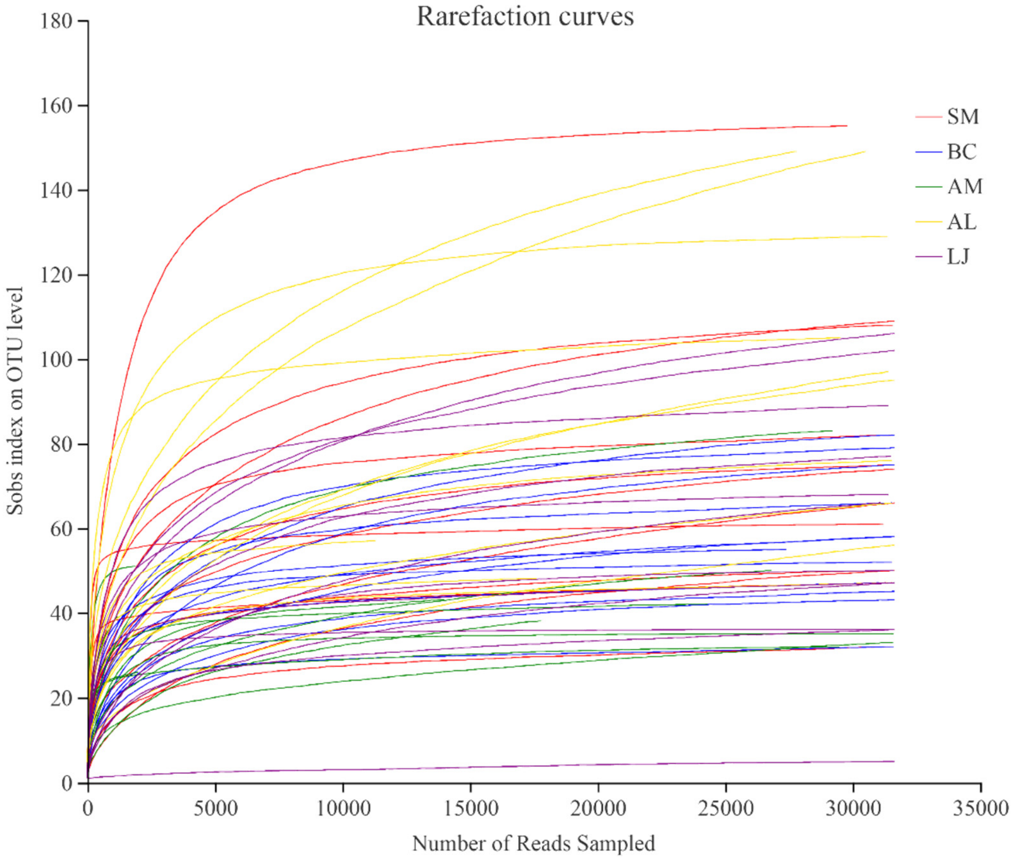

Figure S1. Rarefaction curves for the observed endophytic fungal operational taxonomic units (OTUs). Note: rarefaction curves were assembled showing the number of OTUs, as defined at the 97% sequence similarity cut-off in MOTHUR, relative to the number of total sequences. Abbreviations—SM: *Salvia miltiorrhiza*; BC: *Bupleurum chinense*; AM: *Astragalus membranaceus*; AL: *Atractylodes lancea*; LJ: *Lonicera japonica*.

Table S2. The ten keystone species in the fungal networks for each plant tissue's niches and seasons.

	Order	Genus	OTU	Degree	+	−	Abundance (%)
Root	Glomerales	*Glomus*	301	14	14	0	0.0009
	Saccharomycetales	*Dipodascus*	564	14	5	9	0.0012
	Glomerales	unclassified	298	11	11	0	0.0040
	Glomerales	unclassified	335	11	11	0	0.0017
	Glomerales	*Rhizophagus*	562	11	11	0	0.0083
	Glomerales	unclassified	272	10	10	0	0.0010
	O_unclassified_p_Glomeromycota	unclassified	293	10	10	0	0.0015
	Glomerales	*Rhizophagus*	282	10	10	0	0.0014
	Hypocreales	unclassified	61	9	7	2	0.0012
	Hypocreales	Gibberella	1154	9	7	2	0.0666

Leaf	Pleosporales	*Phaeosphaeria*	252	40	35	5	0.2738
	Pleosporales	*Setophaeosphaeria*	788	39	34	5	0.9333
	Capnodiales	unclassified	870	35	33	2	2.2505
	Capnodiales	unclassified	868	35	35	0	0.1017
	Capnodiales	*Mycosphaerella*	909	35	35	0	0.0624
	Capnodiales	unclassified	857	34	34	0	0.0401
	Capnodiales	unclassified	763	34	33	1	0.1666
	Pleosporales	unclassified	883	34	30	4	0.1123
	Tremellales	*Hannaella*	798	34	31	3	0.0850
	Pleosporales	*Coniothyrium*	666	34	30	4	0.0801
June	Glomerellales	unclassified	165	27	23	4	0.0034
	Hypocreales	*Neocosmospora*	25	21	16	5	0.0133
	Cystofilobasidiales	*Tausonia*	175	20	18	2	0.0032
	Hypocreales	*Neocosmospora*	52	19	16	3	0.0037
	Hypocreales	*Fusarium*	114	19	14	5	0.0002
	Hypocreales	*Dactylonectria*	64	17	15	2	0.0123
	Pleosporales	unclassified	172	17	17	0	0.0024
	Auriculariales	*Oliveonia*	90	16	16	0	0.0003
	Eurotiales	*Aspergillus*	1220	16	4	12	0.0018
	Hypocreales	*Gibberella*	82	15	11	4	0.0161
November	Glomerales	*Glomus*	368	17	17	0	0.0004
	Capnodiales	unclassified	868	17	16	1	0.1017
	Capnodiales	*Cercospora*	885	16	16	0	0.0394
	Glomerales	*Rhizophagus*	282	16	16	0	0.0008
	Glomerales	*Glomus*	301	15	15	0	0.0005
	Capnodiales	unclassified	763	15	14	1	0.1666
	Venturiales	*Ochroconis*	786	14	14	0	0.0034
	Glomerales	*Rhizophagus*	562	14	14	0	0.0046
	Capnodiales	*Sphaerulina*	911	14	14	0	0.0005
	Glomerales	*Glomus*	369	14	14	0	0.0006
Total	Capnodiales	unclassified	763	11	11	0	0.0007
	Helotiales	*Articulospora*	835	11	11	0	0.0012
	Capnodiales	unclassified	721	10	11	0	0.0246
	Venturiales	*Ochroconis*	786	10	10	0	0.0034
	Hypocreales	*Dactylonectria*	64	9	9	0	0.0123
	Capnodiales	*Cercospora*	885	9	9	0	0.0394
	O_unclassified_p_Glomeromycota	unclassified	293	9	9	0	0.0008
	Pleosporales	*Phaeosphaeria*	252	9	9	0	0.0018
	Pleosporales	*Setophaeosphaeria*	788	8	8	0	0.0042
	Chaetothyriales	unclassified	397	8	8	0	0.0066

Note: The degrees are characterized by fungal connections, where +/− indicates positive or negative correlation. Abundance describes the number of OTUs in a group as a proportion of all OTUs (e.g., Abundance $_{OTU301}$% = (OTU301 of abundance/abundance of fungal OTU on the root) × 100).
